# Transactions of the Medical Society of the State of Pennsylvania

**Published:** 1860-05

**Authors:** 


VIII.—*Transactions of the Medical Society of the State of Pennsylvania at its Eleventh Session, held in Philadelphia, June,* 1859. Philadelphia, 1859, pp. 120.

THE results of the session of the State Medical Society in Philadelphia in June last, are recorded in the pamphlet before us. One of the main features of the reports, apart from those presented by county medical societies, was the remarks of Dr. Condie, presented on the second day of the session, on behalf of the committee appointed "to procure a supply of vaccine virus from the original source." His efforts to distribute it for general use were greatly impeded by the fact that the expense and labor incurred in obtaining the virus recently from the cow, and in carefully testing its effects by inserting it in the arms of healthy children, were too onerous to enable a single individual to supply a sufficient amount of recent virus for the wants of the entire profession throughout the State, or any very considerable proportion of it. The Minutes of the meeting occupy but a small portion of the Transactions; the Address of the President, Dr. Smith Cunningham, of Beaver County, and the Reports of County Medical Societies, constituting the main bulk of the pamphlet.

The subject of the Address of the President is, "The period of human utero-gestation, with regard to the errors generally entertained on this subject by those not belonging to the medical profession; their effects upon society; and the remedy." He refers to the little latitude in this matter allowed to women by the community, the immutable nine months being fixed by the common mind as the period of gestation. The medico-legal importance of protracted cases is also referred to, as well as the effects of anticipation of the usual period, especially when occurring in primiparæ. These are the principal points in the Address of Dr. Cunningham, who, at the outset, very properly and modestly declares that, on any subject proper for a discourse before a medical society, it is impossible to say anything that the members do not know; an idea the reverse of that generally entertained by speakers on similar occasions.

We next have presented a "Form of County Reports to the Medical Society of the State of Pennsylvania," which, if adopted, would lead to a more simple method of classifying the information which such reports supply, as well as to a more accessible mode of comparison of the sanitary condition and general characteristics of different portions of the State. The prominent points are, "The Causes which Modify the Health of the County," embracing the subjects of locality, hydrography or drainage, topography, geology, and meteorology; "Mortuary Tables," under which head are included mortality, causes assigned for death, and quarterly tables, showing the whole number of deaths of white and of colored persons at various ages; "Prevalent Diseases," including epidemics and endemics of the year, fevers, and other diseases; and "Miscellaneous Matters," such as the effects of indigenous plants, and of new remedies, facts of interest in surgery and obstetrics, and notices of members deceased during the year.

These would be interesting points, if properly arranged, but we fear few will have the industry to carry out all the details of the tables satisfactorily. The reports which follow certainly attempt occasionally to preserve the order, but at times too briefly. Mortuary tables are not kept in some of the counties, and it is impossible even to approximate the number who have died. Of the interesting cases described, it is not expedient here to allude to any particularly; their history may be found condensed in the pages of the Transactions. The "Obituary Notices" are commemorative of the medical services of Dr. James Anderson, of Montgomery County, Dr. Wm. R. Howe, of Perry County, and of Drs. John Kearsely Mitchell, John H. Weir, and Gavin Watson, of Philadelphia.

The Society meets again in our city on the second Wednesday of June next. We trust that useful results may flow from its deliberations. From the disconnected reports of disease and mortality in various regions of the State, as of the prevalence of malignant scarlatina or measles in one county, and of phthisis or typhoid fever in another, but little can be learned of any general condition of health or disease throughout the State. Some one, who possesses the ability to attempt classification and the industry to accomplish it, might confer a real benefit upon those who do not wish to take the trouble upon themselves, and a useful table of comparison would thus at once exhibit the sanitary condition of different localities.

